# LINC2781 enhances antiviral immunity against coxsackievirus B5 infection by activating the JAK-STAT pathway and blocking G3BP2-mediated STAT1 degradation

**DOI:** 10.1128/msphere.00062-25

**Published:** 2025-07-08

**Authors:** Jiayu Zhang, Jing Li, Yonghan Luo, Timothy J. Mahony, Jingru Gao, Yanchun Wang, Xiaotao Yang, Fan Yang, Xia Ou, Jihong Zhang, Heng Yang, Wei Chen

**Affiliations:** 1 Medical School, Kunming University of Science and Technology 47910, Kunming, Yunnan Province, China; 2 Second Department of Infectious Disease, Kunming Children’s Hospital, Kunming, Yunnan, China; 3 Queensland Alliance for Agriculture and Food Innovation, The University of Queensland 1974, Brisbane, Australia; 4 College of Agriculture and Life Sciences, Kunming University 162634, Kunming, Yunnan Province, China; Instituto de Biotecnologia/UNAM, Cuernavaca, Morelos, Mexico

**Keywords:** coxsackievirus B5 (CVB5), long non-coding RNAs (lncRNAs), LINC2781; GTPase-activating protein SH3 domain-binding protein 2 (G3BP2), STAT1

## Abstract

Coxsackievirus B5 (CVB5) is a primary causative agent of hand, foot, and mouth disease (HFMD), and some cases are also associated with severe complications through invasion of the central nervous system, resulting in death. Currently, there are no specific antiviral drugs or effective vaccines available for CVB5. Long non-coding RNAs (lncRNAs) have been shown to play significant roles in various diseases. In our research, we identified a novel lncRNA, LINC2781, which is significantly upregulated during CVB5 infection of SH-SY5Y cells. Characteristic analysis showed that LINC2781 is mainly located in the cytoplasm of infected cells, with significantly higher expression in the intestines and the spleen of CVB5-infected mice. Functional studies revealed that LINC2781 activates the JAK-STAT pathway via STAT1, promoting the expression of interferon-stimulated genes (ISGs) and inhibiting CVB5 replication. Mechanistically, LINC2781 directly binds to GTPase-activating protein SH3 domain-binding protein 2 (G3BP2), preventing G3BP2-mediated degradation of STAT1 through ubiquitination. *In vivo*, LINC2781 was shown to reduce the susceptibility of BALB/c mice to viral infection and alleviate viral-induced damage in both the intestines and the spleen. Clinical samples further confirmed a strong correlation between the expression of LINC2781 and CVB5 infection. Our findings demonstrate that CVB5-induced LINC2781 enhances STAT1 activation by blocking the suppressive effects of G3BP2 on immune responses, providing a potential foundation for developing antiviral therapies targeting the lncRNA.

**IMPORTANCE:**

We investigate the role of lncRNA in virus-host interactions and identify a novel cytoplasmic lncRNA, LINC2781, whose expression is upregulated following CVB5 infection. LINC2781 specifically binds to G3BP2, preventing G3BP2 from degrading STAT1, thereby activating the JAK-STAT pathway, promoting the expression of ISGs, and ultimately inhibiting viral replication. Meanwhile, a strong correlation exists between the expression of LINC2781 and CVB5 infection in cells and clinical samples.

## INTRODUCTION

Coxsackievirus group B type 5 (CVB5), a member of the *Enterovirus* genus, is responsible for the widely distributed illness, hand, foot, and mouth disease (HFMD). HFMD is particularly prevalent in Asia, affecting millions of children annually and often resulting in public health challenges (1). CVB5 infection typically manifests as papulovesicular rashes on the buccal mucosa and hands, soles, and buttocks, often presenting as a self-limiting acute febrile disease (2). However, it is also associated with severe complications due to infection of the central nervous system, such as aseptic meningitis, acute flaccid paralysis, and brainstem encephalitis, which may result in severe sequelae and even death (3). Currently, symptomatic treatment is limited to patients, as specific antiviral drugs and preventive vaccines are unavailable. CVB5 belongs to the *Picornaviridae* family and has a 7,400 bp positive-sense single-stranded RNA genome encoding four structural proteins (VP1, VP2, VP3, and VP4) and seven non-structural proteins (2A, 2B, 2C, 3A, 3B, 3C, and 3D) (4).

Long non-coding RNAs (lncRNAs) are transcripts longer than 200 nucleotides that lack an open reading frame (ORF) and are considered unlikely to be translated into polypeptides. They regulate various biological processes, including epigenetic modifications, transcription and post-transcriptional gene regulation, and translation (5). Although the roles of lncRNAs in cancer and autoimmune diseases are well-documented (6–8), their involvement in viral pathogenesis, particularly in modulating host immune responses, is still an emerging area of research. The evidence, to date, suggests that host cells utilize lncRNAs to regulate multiple aspects of innate immunity, including cell development, signaling pathways, and gene expression, ultimately influencing viral replication and survival (9–11). Upon viral infection, pattern-recognition receptors (PRRs) detect pathogen-associated molecular patterns (PAMPs) derived from viral products, triggering the activation of type I interferon (IFN-I). This activation initiates the JAK-STAT signaling pathway, increasing the expressions of interferon-stimulated genes (ISGs), which serve as the primary line of host defense against pathogens. LncRNAs have been implicated in regulating multiple steps of the IFN/JAK-STAT signaling pathway (12). Our previous research revealed that LINC1392 regulates melanoma differentiation-associated gene 5 (MDA5) by interacting with ELAV-like RNA-binding protein 1 (ELAVL1), inhibiting CVB5 infection (13). Additionally, lncLrrc55-AS was found to bind to phosphatase methylesterase 1 (PEM-1), preventing the suppression of IRF3 and promoting its phosphorylation and signaling (14). Moreover, Influenza A virus (IAV)-induced lncRNA TSPOAP1-AS1 accumulates in the nucleus and inhibits IFN transcription and expression of ISGs, thereby promoting viral replication (15). However, how host lncRNAs are targeted and utilized by the virus to regulate the JAK-STAT immune response remains poorly understood.

In our previous work, we used RNA sequencing to reveal the characteristics of lncRNA expression in CVB5-infected human neuroblastoma cells (SH-SY5Y), revealing potential regulatory roles of lncRNAs during infection (16). However, evidence of the essential functions of lncRNAs in host-CVB5 interactions remains limited, particularly for *in vivo* models and clinical samples. Herein, we identified the host lncRNA, referred to as LINC2781, which is highly inducible during CVB5 infection in SH-SY5Y cells. LINC2781 directly binds to GTPase-activating protein SH3 domain-binding protein 2 (G3BP2), thereby blocking its interaction with STAT1 and preventing G3BP2-mediated ubiquitination of STAT1. This enhances STAT1 activity and promotes the expression of interferon-stimulated genes (ISGs), thereby inhibiting CVB5 replication. In a mouse model, LINC2781 inhibited viral infection and reduced pathological changes in the intestines and spleen of CVB5-infected mice. Also, clinical samples showed a strong correlation between the expression of LINC2781 and CVB5 infection. Together, these findings suggest that LINC2781 acts as a positive regulator of innate antiviral immunity, providing the foundation for the development of clinical treatments for CVB5 infection.

## RESULTS

### Characteristic of LINC2781 in CVB5-infected SHSY-5Y cells

**Fig 1:**
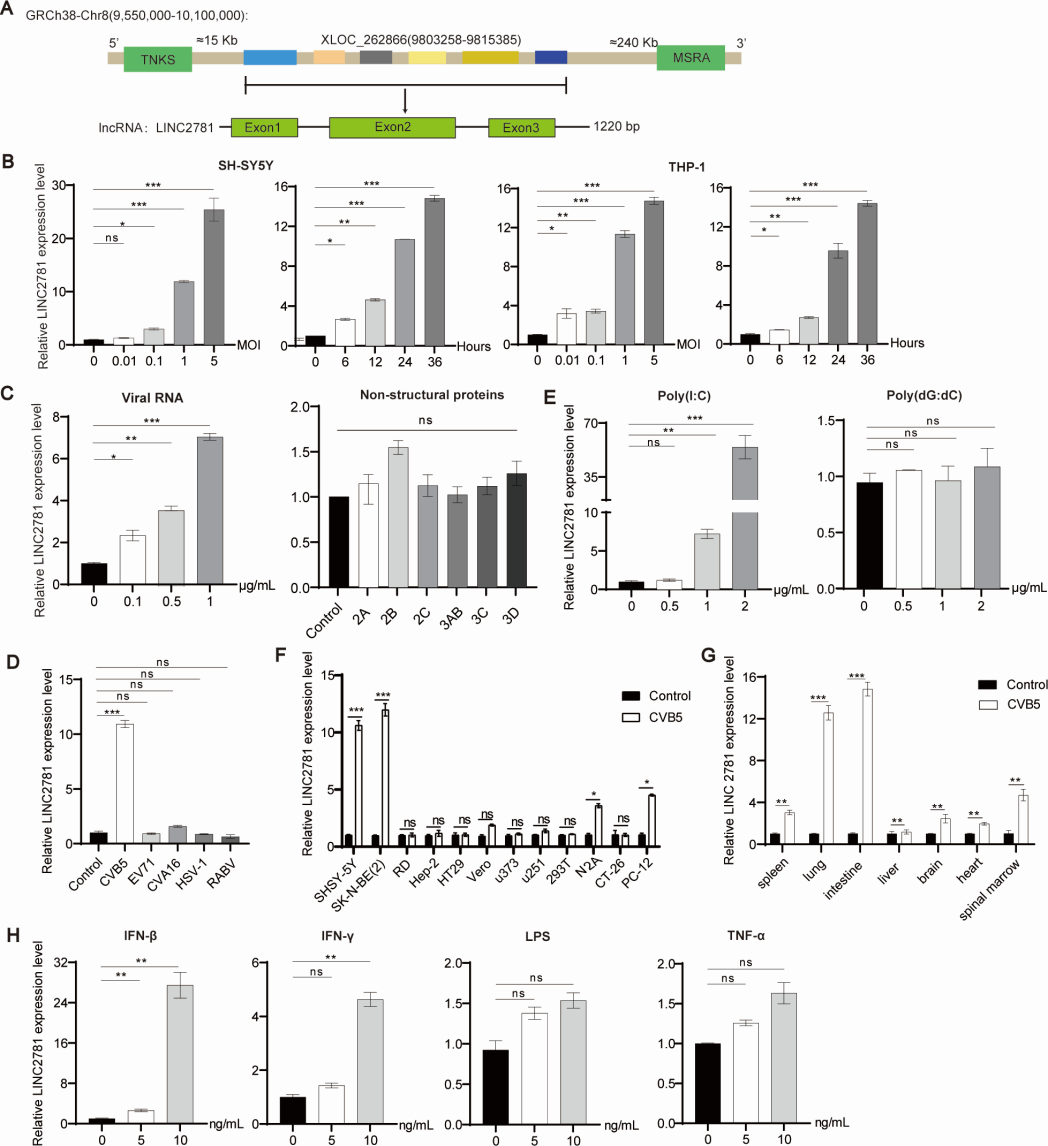
The characterization of LINC2781. (A) A schematic representation of the human LINC2781 transcribed from the XLOC_262866 gene; (B) SH-SY5Y and THP-1 cells were infected with CVB5 at increasing MOIs for 24 h or CVB5 (MOI = 1) for the indicated time intervals. The expression of LINC2781 was measured by RT-qPCR; (C) SH-SY5Y cells were transfected with increasing amounts of CVB5 RNA or plasmids (2 µg) encoding viral nonstructural proteins. The expression of LINC2781 was measured by RT-qPCR; (D) SH-SY5Y cells were infected with CVB5, CVA16, EV71, HSV-1, and RABV (MOI = 1) for 24 h. The expression of LINC2781 was measured by RT-qPCR; (E) SH-SY5Y cells were stimulated with different amounts of poly (I:C) or poly (dG:dC) for 24 h. The expression of LINC2781 was measured by RT-qPCR; (F) Various cell lines were infected with CVB5 (MOI = 1) for 24 h. The expression of LINC2781 was measured by RT-qPCR; (G) Three-day-old BALB/c mice were infected with CVB5 (20 LD50) for 10 days. The expression of LINC2781 was measured by RT-qPCR. (H) SH-SY5Y cells were treated with increasing amounts of IFN-β, IFN-γ, LPS, and TNF-α for 24 h. The expression of LINC2781 was measured by RT-qPCR. Biologically independent experiments (*n* = 3) were conducted, and all data were shown as mean ± SD. Student’s *t*-test was used to detect significant differences, with *P* ≤ 0.05 (*), *P* ≤ 0.01 (**), *P* ≤ 0.001 (***), and ns for no significant difference.

Analysis of RNA-seq data from SH-SY5Y cells infected with CVB5 identified the top 10 differentially expressed lncRNAs (indicated by a *P*-value ≤ 0.05 and a fold change >2) (Fig. S1A). Validation of these results by RT-qPCR showed that LINC2781 was the most differentially expressed lncRNA in CVB5-infected SH-SY5Y cells compared with uninfected cells (Fig. S1B). Further bioinformatic analysis revealed that LINC2781 is a long intergenic non-coding RNA transcribed from XLOC_262866 (9803258-9815385) on chromosome 8q. It consists of three exons with a full length of 1,220 bp (Fig. 1A). According to the PhylocSF prediction, LINC2781 lacks any detectable protein-coding potential (Fig. S2). To assess interspecies sequence variation, we conducted a sequence comparison of LINC2781 using the UCSC. The analysis showed a high degree of sequence identity between LINC2781 transcripts and genes in humans, primates, and rodents (Fig. S3), suggesting that LINC2781 may play a conserved functional role across these species.

Further investigations demonstrated that the expression of LINC2781 was highly inducible in both SH-SY5Y and THP-1 cells in a dose- and time-dependent manner following CVB5 infection (Fig. 1B). Additionally, SH-SY5Y cells were stimulated with viral components, including the viral genome and nonstructural proteins. The results revealed that the significantly increased expression of LINC2781 was only induced by the viral genome and not the nonstructural proteins (Fig. 1C). To better understand the kinetics of the expression of LINC2781 in response to viral infections, we infected SH-SY5Y cells with various viruses such as the main pathogen of HFMD virus strains (EV71 and CVA16), HSV-1, and RABV, which are associated with neurological symptoms and are suitable for infecting SH-SY5Y cells. Interestingly, the expression of LINC2781 was only significantly increased by CVB5 infection (Fig. 1D), consistent with the observation that LINC2781 is significantly induced only by poly (I:C) (Fig. 1E). Analyses of SH-SY5Y cells and nine other cell lines following infection with CVB5 resulted in significantly increased expression of LINC2781 expressed in the neuroblastoma cell line-derived SH-SY5Y, SK-N-BE (2), N2A, and PC-12 cells (Fig. 1F). The expression of LINC2781 was also widely expressed in various organs of mice infected with CVB5, with the highest increases detected in the intestines and lungs (Fig. 1G). These findings suggest that LINC2781 might be closely associated with the clinical symptoms that arise from CVB5 infections. Finally, SH-SY5Y cells were stimulated with IFN-β, IFN-γ, LPS, and TNF-α. The RT-qPCR analyses showed that the expression of LINC2781 was significantly induced by 10 ng of IFN-β and IFN-γ treatments (Fig. 1H). Therefore, these findings suggest that LINC2781 may be involved in the antiviral immune response during CVB5 infection, warranting further investigation into its specific role in regulating host-pathogen interactions.

### LINC2781 facilitates STAT1-mediated innate immune response to suppress CVB5 replication *in vitro*

**Fig 2:**
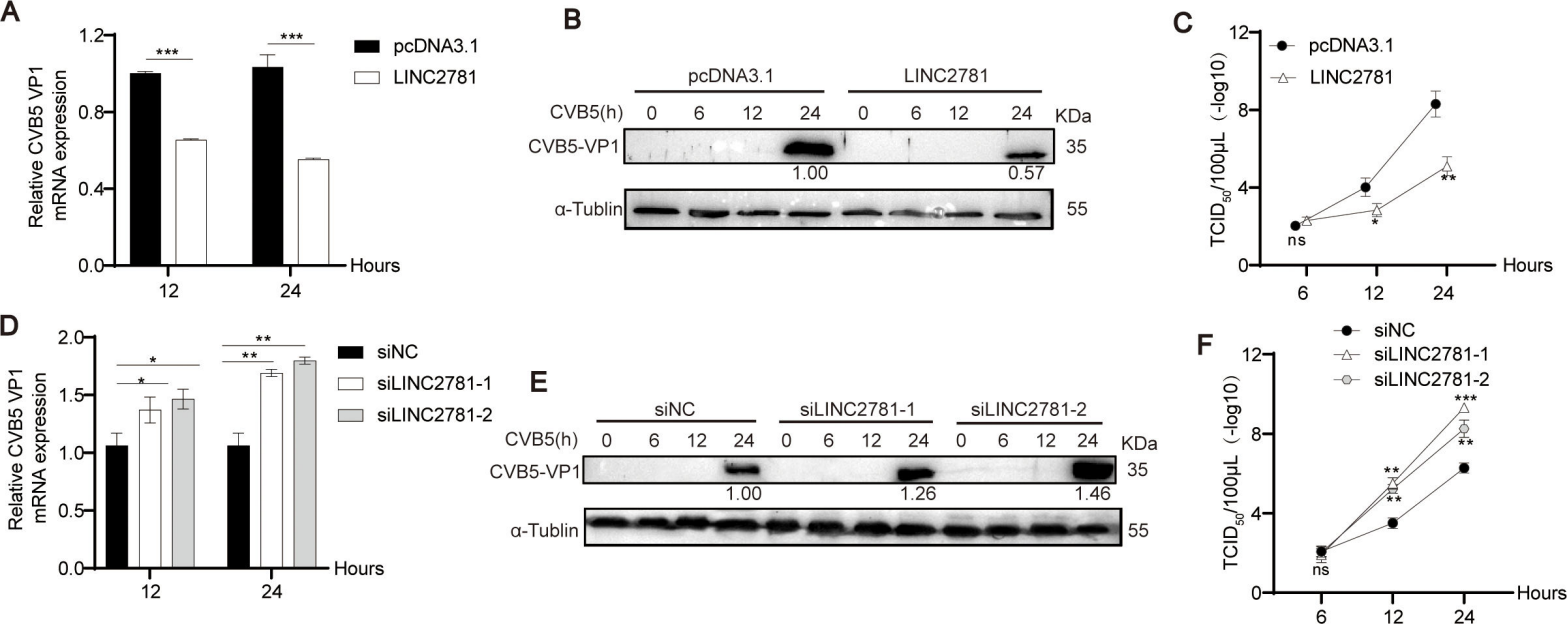
LINC2781 inhibits CVB5 replication. (A through C) LINC2781-overexpressing plasmid (LINC2781) or empty vector (pcDNA3.1) was transfected into SH-SY5Y cells, followed by infection with CVB5 (MOI = 1) at 24 h post-transfection. Cells and supernatants were harvested at 6, 12, or 24 h post-infection (hpi). The expression of CVB5 VP1 mRNA was measured by RT-qPCR (**A**), the expression of CVB5 VP1 protein was measured by western blotting (**B**), and the CVB5 titers were measured by TCID50 assay (**C**); (D through F) siLINC2781-1, siLINC2781-2, or empty vector (siNC) was transfected into SH-SY5Y cells, followed by infection with CVB5 (MOI = 1) at 24 h post-transfection. Cells and supernatants were harvested at 6, 12, or 24 hpi. The expression of CVB5 VP1 mRNA was measured by RT-qPCR (**D**), the expression of CVB5 VP1 protein was measured by western blotting (**E**), and the CVB5 titers were measured by TCID50 assay (**F**). Biologically independent experiments (*n* = 3) were conducted, and all data were shown as mean ± SD. Student’s *t*-test was used to detect significant differences, with *P* ≤ 0.05 (*), *P* ≤ 0.01 (**), *P* ≤ 0.001 (***), and ns for no significant difference. The band intensity of proteins was quantified, and the ratios of the target protein to α-Tubulin were shown.

To further investigate the effects of LINC2781 during CVB5 infection, we successfully constructed the LINC2781-overexpressing plasmid by inserting LINC2781 coding sequence into pcDNA3.1 vector (Fig. S4A). The LINC2781-overexpressing plasmid was transfected into SH-SY5Y cells followed by infection with CVB5. The results demonstrated that LINC2781 inhibited the replication of CVB5 VP1 at both the mRNA and protein levels (Fig. 2A and B). Additionally, the TCID50 assay confirmed that overexpression of LINC2781 significantly reduced viral titers (Fig. 2C). In agreement with these results, when siRNA-mediated knockdown of LINC2781 was performed (Fig. S4B), significant increases in the replication of CVB5 were detected (Fig. 2D through F). Together, these findings suggest that LINC2781 has the potential to suppress the replication of CVB5.

**Fig 3:**
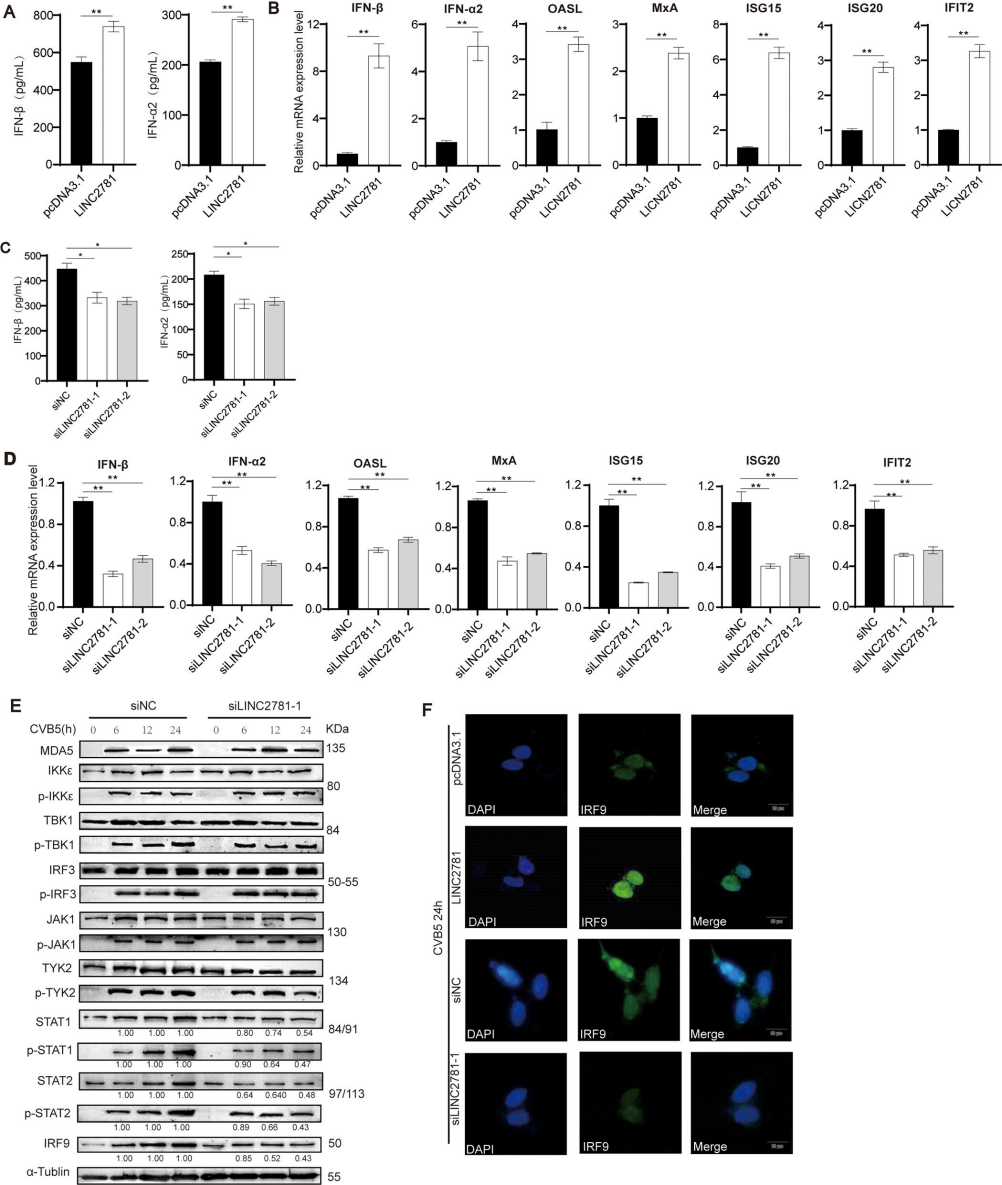
LINC2781 activates ISG expression and the JAK-STAT pathway via STAT1 activation. (A and B) LINC2781-overexpressing plasmid (LINC2781) or empty vector (pcDNA3.1) was transfected into SH-SY5Y cells, followed by infection with CVB5 (MOI = 1) at 24 h post-transfection. Cells and supernatants were harvested at 24 hpi. The secretion of IFN-I was measured by ELISA (**A**), and the expression of IFN-I and ISGs mRNA was measured by RT-qPCR (**B**); (C and D). siLINC2781-1, siLINC2781-2, or empty vector (siNC) was transfected into SH-SY5Y cells, followed by infection with CVB5 (MOI = 1) at 24 h post-transfection. Cells and supernatants were harvested at 24 hpi, the secretion of IFN-I was measured by ELISA, (**C**) and the expressions of IFN-I and ISGs mRNA were measured by RT-qPCR (**D**); (E) siLINC2781-1 or empty vector (siNC) was transfected into SH-SY5Y cells, followed by infection with CVB5 (MOI = 1) at 24 h post-transfection. Cells were harvested at 6, 12, and 24, and the expression of IFN/JAK-STAT pathway proteins was measured by western blotting; (F) LINC2781-overexpressing plasmid (LINC2781), siLINC2781-1, or empty vector was transfected into SH-SY5Y cells, followed by infection with CVB5 (MOI = 1) at 24 h post-transfection. Cells were harvested at 24 hpi, and the location and expression of IRF9 were measured by immunofluorescence. Biologically independent experiments (*n* = 3) were conducted, and all data were shown as mean ± SD. Student’s *t*-test was used to detect significant differences, with *P* ≤ 0.05 (*), *P* ≤ 0.01 (**), *P* ≤ 0.001 (***), and ns for no significant difference. The band intensity of proteins was quantified, and the ratios of the target protein to α-Tubulin were shown.

To further explore the role of LINC2781 in antiviral response, we examined the production of IFN-I and the expression of effector genes. Overexpression of LINC2781 in SH-SY5Y cells significantly increased the secretion of IFN-α and IFN-β (Fig. 3A), as well as the mRNA expression of IFN-I and ISGs (Fig. 3B). However, the mRNA expression of inflammatory factors remained unchanged (Fig. S5A). Consistent effects were observed when knockdown of LINC2781 in cells followed by CVB5 infection with significantly less secretion of IFN-α and IFN-β, and expression of IFN-I and ISGs transcripts (Fig. 3C and D; Fig. S5B). Next, we investigated the impact of LINC2781 on the IFN/JAK-STAT pathway during viral infection. We observed that the knockdown of LINC2781 significantly reduced the expression of STAT1/p-STAT1 and the downstream JAK-STAT pathway IRF9 protein in CVB5-infected cells after 24 h post-infection (Fig. 3E). Additionally, immunoflu analysis with an anti-IRF9 antibody confirmed that overexpression of LINC2781 promoted the translocation of activated IRF9 into the nucleus (Fig. 3F). Taken together, all data demonstrate that LINC2781 is crucial in promoting the STAT1-mediated JAK-STAT pathway, enhancing the antiviral innate response.

### LINC2781 directly binds to G3BP2 and blocks G3BP2-mediated STAT1 degradation

**Fig 4:**
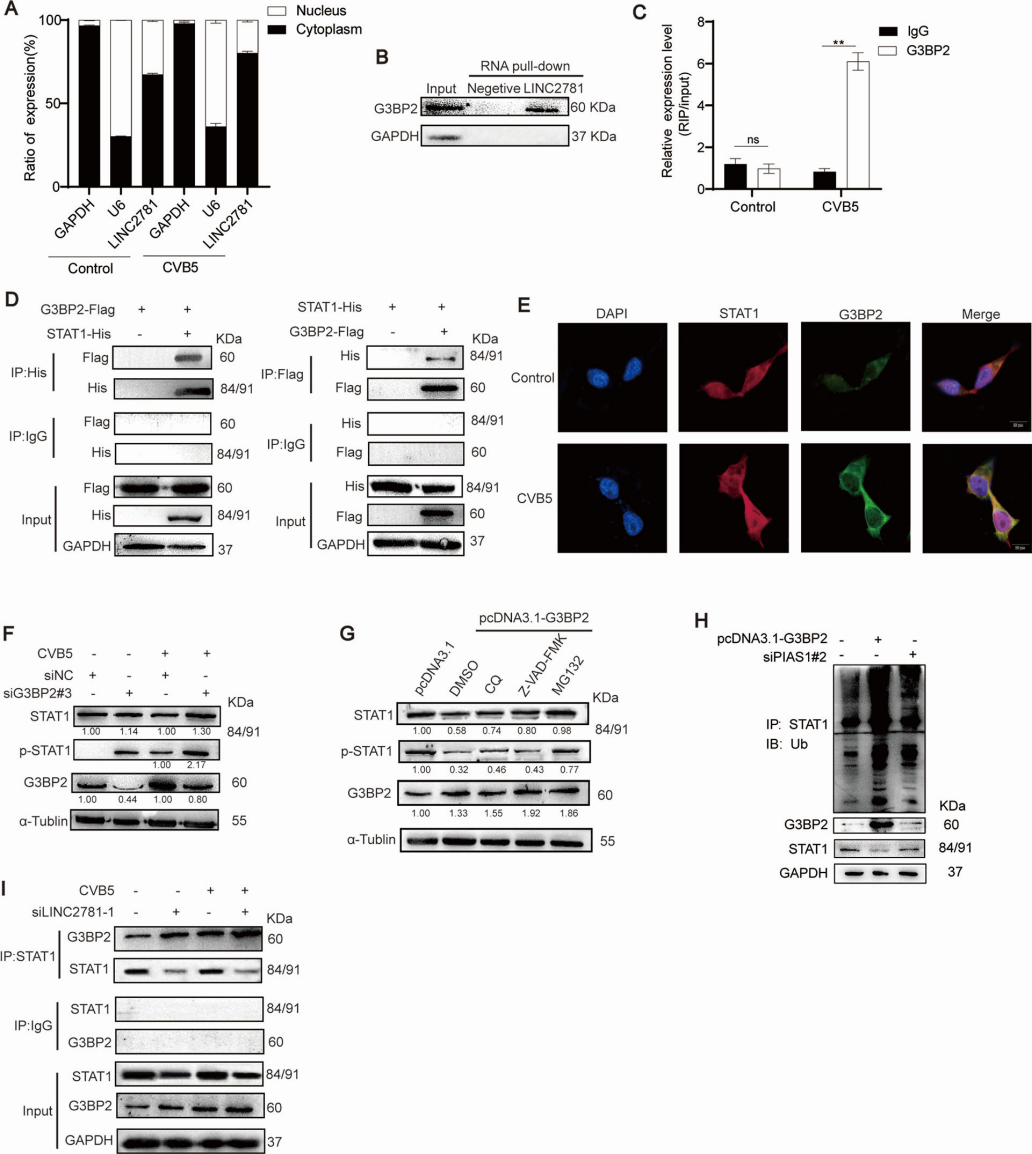
LINC2781 blocks G3BP2-mediated STAT1 degradation. (A) SH-SY5Y cells were infected with CVB5 (MOI = 1), then the cytoplasmic and nuclear fractions were collected and separated at 24 hpi. The expression of LINC2781 in different subcellular fractions was measured by RT-qPCR. GAPDH and U6 mRNA are, respectively, used as cytoplasmic and nuclear controls; (B) RNA pull-down analysis of LINC2781 binding to G3BP2. SH-SY5Y cells were infected with CVB5 (MOI = 1) and harvested at 24 hpi. The biotinylated LINC2781-positive or -negative strand was incubated with magnetic beads to obtain protein-RNA complexes. Complexes were then separated by 10% SDS-PAGE gel and analyzed by G3BP2 western blotting. (C) The binding between LINC2781 and G3BP2 was confirmed by RIP-qPCR. SH-SY5Y cells were infected with CVB5 (MOI = 1) and harvested at 24 hpi. Cell lysates were incubated with G3BP2 antibody (IgG as control) and magnetic beads to obtain the protein-RNA complexes. The total RNA was extracted from complexes, and the expression of LINC2781 was measured by RT-qPCR (%input). (D) pcDNA3.1-G3BP2-2Flag and pcDNA3.1-STAT1-6His were co-transfected into HEK293T cells for 24 h, and the Co-IP analysis was performed to determine the interaction between G3BP2 and STAT1. (E) pcDNA3.1-G3BP2-2Flag and pcDNA3.1-STAT1-6His were co-transfected into SH-SY5Y cells, followed by infection with CVB5 (MOI = 1) at 24 h post-transfection. The location and interaction between G3BP2 and STAT1 were conducted to immunofluorescence. (F) siG3BP2#3 or empty vector (siNC) was transfected into SH-SY5Y cells, followed by infection with CVB5 (MOI = 1) 24 h post-transfection. Cells were harvested at 24 hpi, and the expression of STAT1/p-STAT1 and G3BP2 was measured by western blotting. (G) pcDNA3.1-G3BP2 was transfected into SH-SY5Y cells, followed by infection with CVB5 (MOI = 1) 24 h pos-transfection. Cells were then treated for 48 h with autophagy inhibitor CQ, the caspase inhibitor Z-VAD-FMK, or the proteasome inhibitor MG132. The expression of STAT1/p-STAT1 and G3BP2 was measured by western blotting. (H) pcDNA3.1-G3BP2 and/or siPIAS1#2 were transfected into SH-SY5Y cells, followed by infection with CVB5 (MOI = 1) 24 h post-transfection. Immunoprecipitation analysis was performed to determine the ubiquitination of STAT1. (I) siLINC278-1 or empty vector (siNC) was transfected into SH-SY5Y cells, followed by infection with CVB5 (MOI = 1) 24 h post-transfection. Cells were harvested at 24 hpi, and Co-IP analysis was conducted to determine the interaction between G3BP2 and STAT1. Biologically independent experiments (*n* = 3) were conducted, and all data were shown as mean ± SD. Student’s *t*-test was used to detect significant differences, with *P* ≤ 0.01 (**) and ns for no significant difference. The band intensity of proteins was quantified, and the ratios of the target protein to α-Tubulin were shown.

To elucidate the molecular mechanisms of LINC2781, we analyzed its subcellular distribution by qPCR of nuclear and cytoplasmic fractions. LINC2781 was predominantly located in the cytoplasm after CVB5 infection (Fig. 4A). Neither over-expression nor siRNA-mediated knockdown of LINC2781 did not affect the expression of the genes that are located immediately upstream and downstream from it (Fig. S6). However, the RNA pull-down assay confirmed that LINC2781 does not directly interact with STAT1 (Fig. S7). These results suggest that LINC2781 may exert regulatory effects by binding to cytoplasmic proteins. To identify physically binding proteins in SH-SY5Y cells, LINC2781-associated proteins were separated by gel electrophoresis, and G3BP2 was identified as a candidate LINC2781-binding protein (Fig. S8). We further performed RNA pull-down analysis to confirm the binding association between LINC2781 and G3BP2. The results showed that LINC2781 directly binds to G3BP2 (Fig. 4B). We also carried out the RIP assay to validate the associations and observed the enrichment of LINC2781 in the immunoprecipitated samples using the anti-G3BP2 antibody (Fig. 4C). Moreover, LINC2781 reduced the expression of G3BP2 at both mRNA and protein levels after CVB5 infection (Fig. S9).

Next, to investigate if G3BP2 directly interacts with STAT1, we co-constructed Flag-tagged G3BP2 and His-tagged STAT1 in HEK293T cells. Exogenous co-immunoprecipitation (Co-IP) assays demonstrated an interaction between G3BP2 and STAT1 (Fig. 4D). Immunofluorescence analysis further supported the hypothesis that G3BP2 increasingly binds to STAT1 in the cytoplasm during virus infection (Fig. 4E). To identify the specific binding site on STAT1, Pymol predictions indicated that the primary interaction occurs at the STAT1 Y701 domain, a key phosphorylation site for STAT1 activation (Fig. S10). Furthermore, we used an antibody specifically for STAT1 phosphorylation at Y701 and confirmed that G3BP2 downregulates STAT1 phosphorylation at the STAT1 Y701 at the protein level (Fig. 4F; Fig. S11 and S12). To explore the mechanism of STAT1 degradation, the cells were treated with the inhibitors (Z-VAD-FMK, CQ, and MG132) and then evaluated. The protease inhibitor MG132 restored the expression of STAT1 at the protein level, indicating that G3BP2 downregulates the STAT1 Y701 through the ubiquitination pathway (Fig. 4G). PIAS1, a protein inhibitor of activated STAT1, is a specific suppressor protein involved in the regulation of various transcription factors and ubiquitination. We synthesized siRNA-mediated knockdown of PIAS1 (Fig. S13) and co-transfected it with G3BP2. The results confirmed that G3P2 regulates STAT1 Y701 ubiquitination through PIAS1 (Fig. 4H). Finally, we demonstrated that LINC2781 effectively inhibits the binding of G3BP2 to STAT1 following CVB5 infection (Fig. 4I). Therefore, we conclude that LINC2781 exerts its function by influencing the interaction between G3BP2 and STAT1.

### LINC2781 relies on G3BP2 to exert antiviral effects

**Fig 5:**
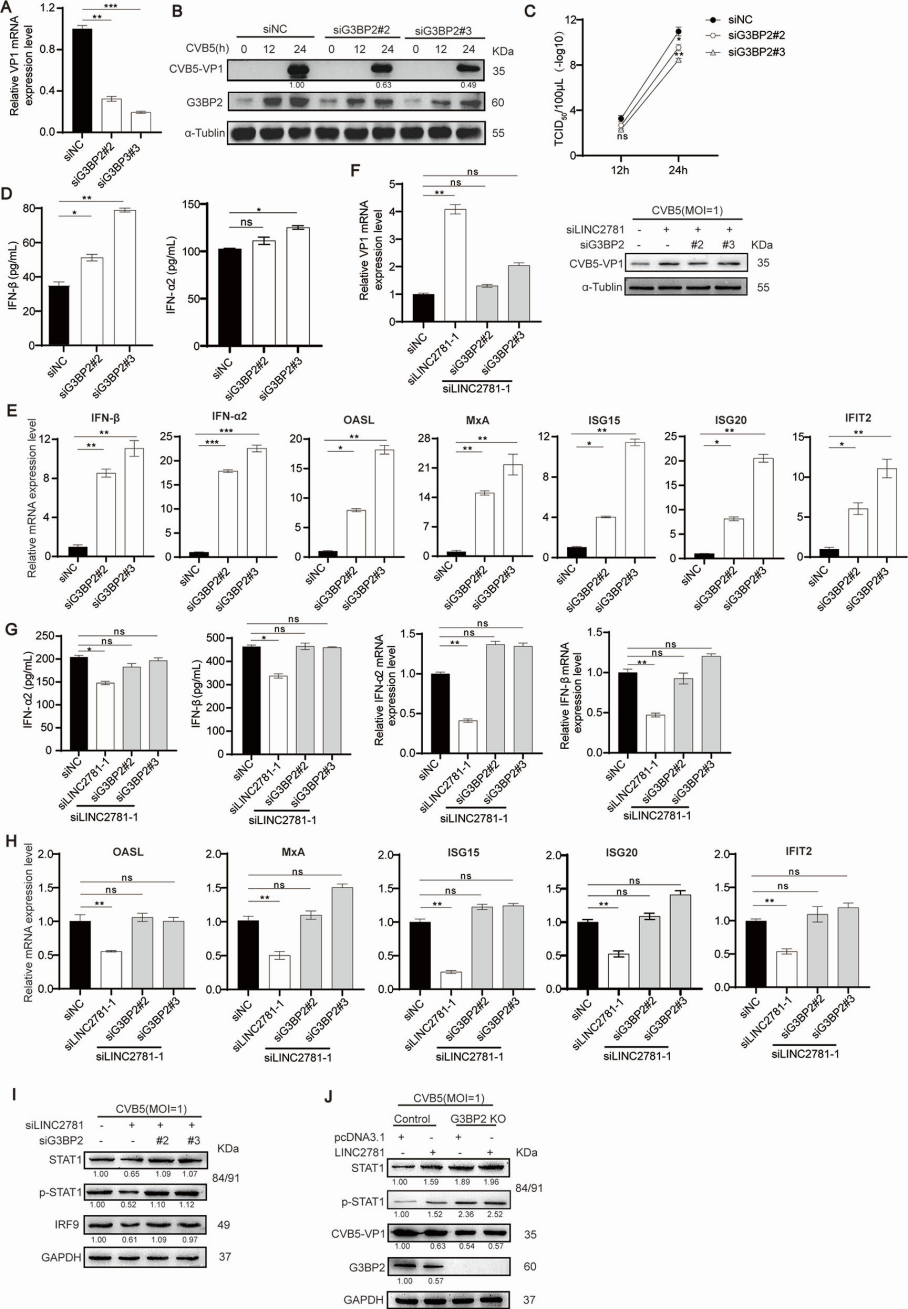
LINC2781 relies on G3BP2 to exert antiviral effects. (A through E) siG3BP2#2, siG3BP2#3, or siNC was transfected into SH-SY5Y cells, followed by infection with CVB5 (MOI = 1) at 24 h post-transfection. Cells and supernatants were harvested at 24 hpi. The expression of CVB5 VP1 mRNA was measured by RT-qPCR (**A**). The expression of CVB5 VP1 protein was measured by western blotting (**B**). The CVB5 titers were measured by TCID50 assay (**C**). The secretion of IFN-I was measured by ELISA (**D**). The expression of IFN-I and vital ISGs mRNA was measured by RT-qPCR (**E**). F-I. siG3BP2#2, siG3BP2#3, and siLINC2781-1 were co-transfected into SH-SY5Y cells, followed by infection with CVB5 (MOI = 1) at 24 h post-transfection. Cells and supernatants were harvested at 24 hpi. The expression of CVB5 VP1 was measured by RT-qPCR and western blotting (**F**). The expression of IFN-I was measured by ELISA and RT-qPCR (**G**). The expression of vital ISGs mRNA was measured by RT-qPCR (**H**). The expressions of STAT1/p-STAT1 and IRF9 were measured by western blotting (**I**). (J) LINC2781-overexpressing plasmid (LINC2781) or empty vector (pcDNA3.1) was transfected into G3BP2 KO cells, followed by infection with CVB5 (MOI = 1) at 24 h post-transfection. Cells and supernatants were harvested at 24 hpi. The expression of STAT1/p-STAT1, CVB5 VP1, and G3BP2 was measured by western blotting. Biologically independent experiments (*n* = 3) were conducted, and all data were shown as mean ± SD. Student’s *t*-test was used to detect significant differences, with *P* ≤ 0.05 (*), *P* ≤ 0.01 (**), *P* ≤ 0.001 (***), and ns for no significant difference. The band intensity of proteins was quantified, and the ratios of the target protein to α-Tubulin were shown.

G3BP2 is known to regulate virus replication; however, the role of G3BP2 in CVB5 replication remains largely unexplored. siRNA-mediated knockdown of G3BP2 was transfected into SH-SY5Y cells, followed by infection with CVB5. The results demonstrated that the replication of CVB5 was significantly suppressed at 24 hpi (Fig. 5A through C). In addition, IFN-I secretion (Fig. 5D), as well as the expressions of IFN-I and ISGs mRNA (Fig. 5E), was markedly increased, suggesting that G3BP2 could inhibit ISGs and IFN-I expression and potentially promote the replication of CVB5. Next, we examined whether LINC2781 exerts its function by relying on G3BP2. Knockdown of both LINC2781 and G3BP2 in SH-SY5Y cells did not further affect the replication of CVB5 (Fig. 5F), the production of IFN-I (Fig. 5G), or the expression of ISGs (Fig. 5H). Notably, STAT1 and its downstream IRF9 protein levels also recovered as expected (Fig. 5I), supporting the hypothesis that LINC2781 functions through interacting with G3BP2. Finally, we generated G3BP2 knockout HEK293 cells, and the results demonstrated that LINC2781 lost its function in the absence of G3BP2 (Fig. 5J; Fig. S14). Therefore, we conclude that LINC2781 is indispensable for the virus-triggered JAK-STAT pathway via STAT1 by interacting with G3BP2.

### The 5’-terminal of LINC2781 is the main functional site

**Fig 6:**
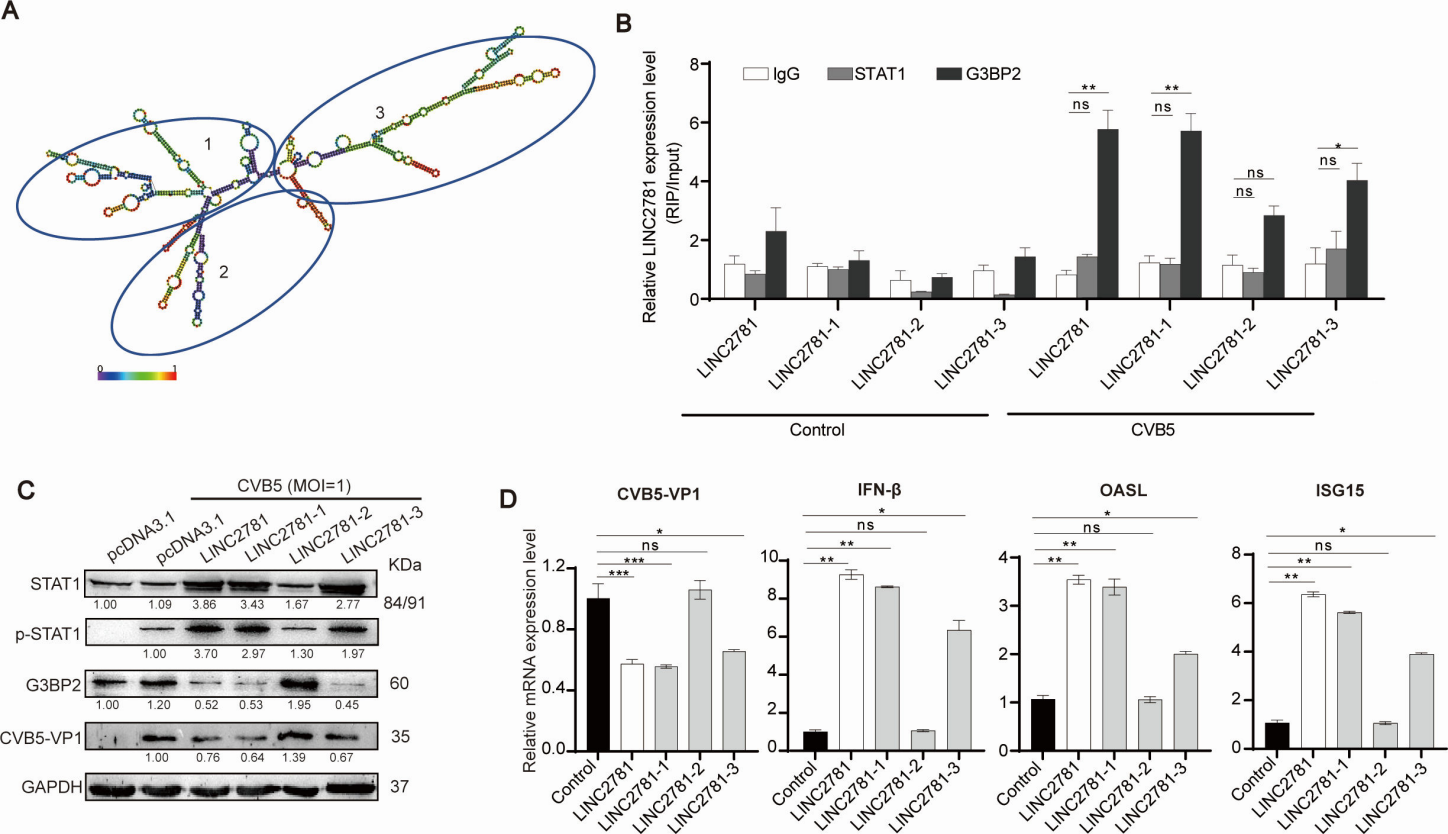
The 5’-terminal of LINC2781 is the main functional site. (A) The secondary structure of LINC2781 was analyzed by RNA-fold. Base pairing probabilities are color-coded on a scale from 0 (blue) to 1 (red). (B-D) LINC2781-overexpressing plasmid (LINC2781), its truncated functional domain plasmids (LINC2781-1, LINC2781-2, and LINC2781-3) or an empty vector (pcDNA3.1) was transfected into SH-SY5Y cells, followed by infection with CVB5 (MOI = 1) 24 h post-transfection. Cell lysates were incubated with the G3BP2 antibody (IgG as control) and magnetic beads to obtain the protein-RNA complexes. The total RNA was extracted from complexes, and the expression of LINC2781 was measured by RT-qPCR (%input). (**B**) The expression of STAT1/p-STAT1, G3BP2, and CVB5 VP1 was measured by western blotting (**C**). The expression of CVB5 VP1, IFN-β, OASL, and ISG15 mRNAs was measured by RT-qPCR (**D**). Biologically independent experiments (*n* = 3) were conducted, and all data were shown as mean ± SD. Student’s *t*-test was used to detect significant differences, with *P* ≤ 0.05 (*), *P* ≤ 0.01 (**), *P* ≤ 0.001 (***), and ns for no significant difference. The band intensity of proteins was quantified, and the ratios of the target protein to α-Tubulin were shown.

The secondary structure of lncRNAs is closely related to their diverse functions. Based on the RNA-fold web server predictions, LINC2781 consists of three major sub-structures with base-paired and hairpin structures (Fig. 6A). To further define the functional domains of LINC2781, we cloned segments corresponding to the three sub-structures (LINC2781_1-430_, LINC2781_431-760_, and LINC2781_761-1220_) into the pcDNA3.1 vector (Fig. S15) and transfected them into SH-SY5Y cells. RIP assay showed that the 5’-terminal domains of LINC2781 (LINC2781_1-430_) might form a spatial structure that is essential for the interaction with G3BP2 (Fig. 6B). Meanwhile, the 5’-terminal of LINC2781 also influenced the replication of CVB5, the expression of STAT1/p-STAT1 and G3BP2, as well as the expression of ISGs mRNA (Fig. 6C and D).

### LINC2781 suppressed CVB5 infection *in vivo*

**Fig 7:**
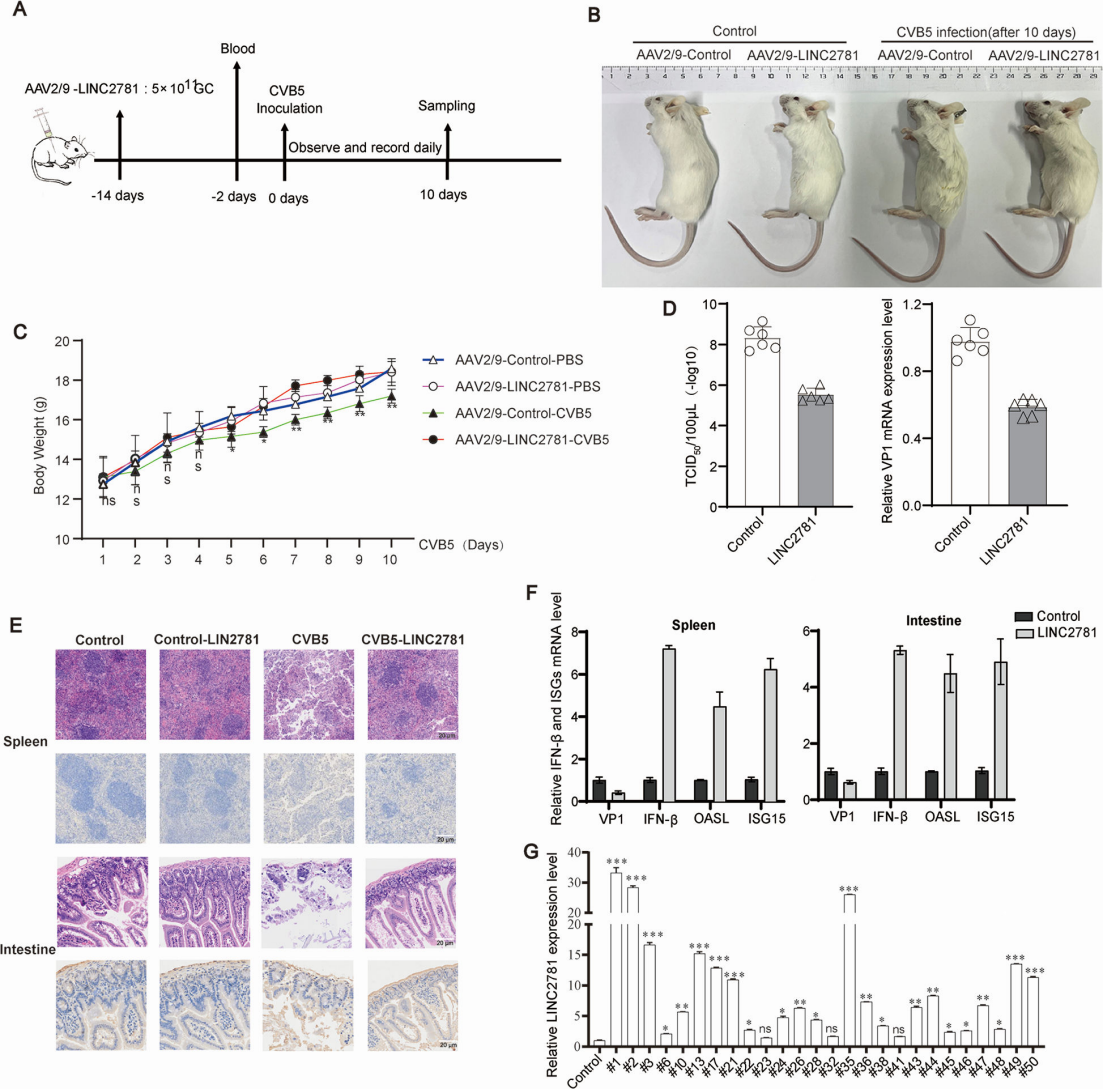
The role of LINC2781 *in vivo*. (A). Schematic diagram of the mice models. Three-day-old BALB/c mice were injected intraperitoneally with AAV2/9-LINC2781 (5 × 10^11^ genomic copies) or AAV2/9 (2 × 10^11^ genomic copies) thrice. Fourteen days later, the mice were infected with CVB5 (20 LD50) by intraperitoneal injection. Samples were collected 10 days post-infection. (B) Appearance characteristics of the mice. (C) Changes in mice body weight. (D) The expression of CVB5 was measured by TCID50 assay and RT-qPCR. (E) Pathological symptoms in the spleen and intestines were assessed by HE staining, and the expression of G3BP2 in the spleen and intestines was measured by IHC. (F) The expression of CVB5 VP1, IFN-β, OASL, and ISG15 mRNAs in the spleen and intestines was measured by RT-qPCR. (G) The expression of LINC2781 in clinical samples was measured by RT-qPCR. Six BALB/c mice were used for the experimental group, and all data were shown as mean ± SD. Student’s *t*-test was used to detect significant differences, with *P* ≤ 0.05 (*), *P* ≤ 0.01 (**), and ns for no significant difference.

To explore the role of LINC2781 during CVB5 infection *in vivo*, we established a mouse model for overexpression of LINC2781 by injecting AAV2/9 -LINC2781. Mice were then infected with CVB5 (20 LD50) by intraperitoneal injection and monitored for 10 days post-infection (Fig. 7A). Fourteen days after AAV2/9-LINC2781 injection, a significant increase in the expression of LINC2781 was detected in the blood of treated mice (Fig. S16). Following intraperitoneal challenge with CVB5, in the LINC2781-overexpressing group, the clinical signs were notably improved compared with the control mice (Fig. 7B), and the body weight was restored similar to the uninfected controls (Fig. 7C). Additionally, the viral loads of CVB5 and the expression of VP1 were reduced in blood in the AAV2/9-LINC2781 treated mice compared with the untreated controls (Fig. 7D). Meanwhile, HE staining revealed significantly reduced lesions in the intestine and spleen of the mice, whereas IHC showed a reduction of the expression of G3BP2 (Fig. 7E), accompanied by increased expression of IFN-β and ISGs (Fig. 7F). These data suggest that LINC2781 suppresses CVB5 infection *in vivo* and improves pathological changes in the intestine and spleen.

Finally, we revealed the relevance of LINC2781 in clinical samples from CVB5-infected patients. The patients were confirmed as having CVB5 infection through RT-qPCR and sequence identification (Table 1; Fig. S17). RT-qPCR analysis of the clinical samples revealed that the expression of LINC2781 was associated with CVB5 infection (Fig. 7G). These results suggest that LINC2781 is correlated with CVB5-infected disease. Together, these findings indicate that LINC2781 could be an effective and safe potential therapeutic agent against CVB5.

**TABLE 1:** Characteristics of clinical samples^*a*^

Patient	CVB5 (TCID50/μL)	Other enterovirus
#1	4.19	/
#2	4.08	/
#3	3.43	/
#4	/	EV71
#5	/	EV71
#6	1.22	/
#7	/	EV71
#8	1.53	/
#9	/	EV71
#10	2.05	/
#11	/	CVA16
#12	/	CVA16
#13	3.41	/
#14	/	EV71
#15	/	EV71
#16	/	EV71
#17	3.30	/
#18	/	EV71
#19	/	CVA16
#20	/	CVA16
#21	3.06	/
#22	1.51	/
#23	0.32	/
#24	2.24	/
#25	/	CVA16
#26	2.33	/
#27	/	EV71
#28	2.03	/
#29	/	EV71
#30	/	EV71
#31	/	EV71
#32	0.33	/
#33	/	EV71
#34	/	EV71
#35	3.93	/
#36	2.43	/
#37	/	EV71
#38	2.68	/
#39	/	EV71
#40	/	EV71
#41	0.36	/
#42	/	CVA16
#43	2.18	/
#44	2.57	/
#45	1.44	/
#46	1.48	/
#47	2.18	/
#48	1.51	/
#49	3.33	/
#50	3.17	/

^*a*^ All the patients were aged 3–5 years. All the patients were diagnosed with herpetic angina or mild HFMD. No other enterovirus detected was indicated by “/”.

## DISCUSSION

The innate antiviral immune response serves as the first line of defense between the host and the virus. To survive infection, the cells employ various mechanisms to overcome virus-induced immune evasion. LncRNAs are critical regulators of IFN-I innate immune responses (17). Identifying key lncRNAs involved in the IFN-I innate antiviral response against CVB5 is essential for advancing our understanding of viral diseases. Previously, our lab identified two lncRNAs involved in the IFN-I pathways, such as LINC1392, which regulated the melanoma differentiation-associated gene 5 (MDA5) by interaction with ELAV-like RNA binding protein 1 (ELAVL1) to inhibit CVB5 infection (13). In this study, we identified LINC2781 as a novel and abundant lncRNA in SH-SY5Y cells, which functions as an anti-CVB5 effector by regulating the JAK-STAT signaling via the activation of STAT1. Together, our findings demonstrate that lncRNAs can selectively target CVB5 infection through IFN-mediated pathways. Further investigation into their functions may uncover new mechanisms by which lncRNAs regulate viral infection and/or act as regulators of innate immunity, potentially adding another layer to our understanding of classic innate immune signaling.

LINC2781 is a highly inducible intergenic lncRNA with significant species homology, induced by CVB5 infection in both SH-SY5Y and THP-1 cells. Additionally, IFN-I (IFN-β and IFN-γ) stimulation induces the expression of LINC2781, indicating an interferon-dependent (18). These fundamental characteristics suggest that further research into LINC2781 is of great significance. Next, we demonstrate that LINC2781 regulates the JAK-STAT pathway by enhancing STAT1 activation, which subsequently promotes the expression of IFN-I and ISGs, thereby inhibiting the replication of CVB5. So far, multiple regulatory mechanisms have been identified at different levels to regulate the IFN-I pathway.

The subcellular localization of lncRNA determines their function (19–21). Cytoplasmic lncRNAs are often involved in post-transcriptional regulation by binding intracellular proteins (22). Our data show that LINC2781 is primarily localized in the cytoplasm and does not directly interact with STAT1, suggesting that its function may be mediated through interactions with RNA-binding proteins. In this study, we demonstrated that G3BP2 binds to LINC2781 in the cytoplasm following CVB5 infection. G3BP2 is a crucial protein involved in the formation of stress granules (SGs) within the cytoplasm. Recent studies indicate that G3BP2 can either restrict viral replication by trapping viral proteins in SGs or facilitate viral evasion of the host immune response, depending on the virus and the cell type (23, 24). Most reports have revealed that the function of G3BP2 depends on the association with the host protein (25, 26). However, we found that in SH-SY5Y cells, G3BP2 regulates CVB5 infection by binding to the host lncRNA-LINC2781, highlighting that G3BP2 can exert multiple antiviral mechanisms in different ways.

Further research revealed that G3BP2 interacts with STAT1, one of the six major isoforms in the STATs family. Members of the STAT family have been linked to cancer development, progression, metastasis, survival, and treatment resistance (27). The importance of STAT-mediated IFN signaling as a key component of antiviral defense is well established. Canonical STAT signaling is initiated by STAT1 phosphorylation, peaking within 30–60 min, during which p-STAT1 accumulates in the nucleus to activate ISGs. The process of STAT1-directed IFN antagonism involves targeting STAT1 for degradation, sequestration, and cytoplasmic retention (28, 29). Variations on these basic themes of STAT inhibition have been reinvented by many viruses to regulate IFN responses. For example, SARS-CoV-2 N protein interacts with STAT1 and STAT2, preventing their nuclear translocation (30). Similarly, RABV *P* protein selectively targets IFN-activated p-STAT1, providing insights into specific antiviral mechanisms (31). DEAD-Box Helicase 5 (DDX5) regulates the IFN response by regulating STAT1 mRNA translation via the resolution of a G-quadruplex (rG4) RNA structure, thereby exerting anti-HBV effects (32). Here, we found that G3BP2 mediates the ubiquitination of STAT1 at Y701 in CVB5-infected cells, inhibiting STAT1 activation and IFN signaling. Specifically, the ubiquitination of STAT1 at Y701 is a crucial modification that diminishes its kinase activity, ultimately impairing the host’s antiviral signaling cascade. Notably, G3BP1, a member of the same family as G3BP2, interacts with the deubiquitinase USP10 through its TFII-like domain (33). Although G3BP2 lacks canonical E3 catalytic domains (RING or HECT), we hypothesize that it may serve as a scaffold to recruit specific E3 ligases to the STAT1 complex, analogous to the G3BP1-USP10 interaction. This proposed mechanism could explain how G3BP2 facilitates STAT1 ubiquitination despite lacking intrinsic E3 activity. This might be a negative feedback mechanism used by the host to avoid an excessive immune response; however, it is leveraged by CVB5 for immune escape.

The diverse functions of lncRNAs are closely related to their secondary structure (34). To clarify the functional sites of LICN2781, we examine the different domains of the lncRNA. The results show that the deletion of nucleotides 1–430 significantly impaired the function of LINC2781, indicating that the 5'-terminal region is critical for its function.

Our research highlights the potential role of LINC2781 in the host response to CVB5 infection. However, further *in vivo* and clinical evidence is required to confirm the putative function of LINC2781. Overexpression of LINC2781 in mice confirmed that LINC2781 inhibits viral replication and alleviates clinical signs in the model. Additionally, our research is the first to report the expression of LINC2781 in clinical samples from CVB5-infected patients, revealing a potentially crucial role for lncRNAs in determining CVB5-induced clinical manifestation. However, since the clinical samples were stored in tubes containing virus-inactivated fluid, it was impossible to accurately quantify the loads of the virus. We performed relative quantification of the viral titers in the samples using a virus standard and confirmed that the expression of LINC2781 is highly correlated with CVB5 infection. The mechanism of LINC2781 still needs further validation in the clinical samples.

Collectively, these data demonstrate LINC2781 directly binds to G3BP2, preventing G3BP2-mediated degradation of STAT1 through ubiquitination, thereby increasing STAT1-mediated JAK-STAT signaling pathway activation, resulting in inhibition of the replication of CVB5. LINC2781, as well as G3BP2 inhibitors or agonists, may represent potential targets for controlling CVB5 infections and the associated HFMD diseases.

## MATERIALS AND METHODS

### Cells and virus

The human neuroblastoma-derived cells (SH-SY5Y and SK-N-BE (2)), human Rhabdomyosarcoma cells (RD), human peripheral blood mononuclear cells (THP-1), human embryonic kidney 293T (HEK 293T), monkey African green kidney cells (Vero), U373 cells, U251 cells, Hep-2 cells, Neuro-2A (N2A) cells, and PC cells were cultured in Dulbecco’s modified Eagle’s medium (DMEM) (11965092, ThermoFisher). HT29 cells were cultured in RPMI 1640 medium (12633012, ThermoFisher). All media were supplemented with 10% (vol/vol) fetal bovine serum (FBS) (A5670701, ThermoFisher). Cells were cultured at 37°C in a humidified atmosphere of 5% CO_2_.

The CVB5 strain (GenBank: MH201081.1) was isolated from a HFMD patient in Kunming, Yunnan Province, China, in 2014. Coxsackievirus A16 (CVA16) strain 74/YN/2016 (GenBank: KY440934.1), enterovirus A71 (EV71) strain RA330/YN/CHN/2009 (GenBank: MK028135.1), human herpes virus 1 (HSV-1) isolate ZW6 (GenBank: KX424525.1KX424525.1), and rabies virus CVS-11 (RABV, ATCC VR 959) were stored in our laboratory. CVB5 was serially diluted from 10^−1^ to 10^−15^ with DMEM, and Vero cells were infected with the serial dilutions. The cytopathic effects (CPE) were recorded for 5–7 days. The 50% Tissue Culture Infectious Dose (TCID50) assay was calculated using the Reed‐Muench method.

### Construction of plasmid and transfection

The sequences of LINC2781 and G3BP2 were cloned into the pcDNA 3.1(+) plasmid to facilitate constitutive overexpression. RNA interference-mediated knockdown of LINC1392, G3BP2, and PIAS1 was facilitated with synthetic siRNA by RiboBio Co., Ltd (Guangzhou, China). The target sequences of the siRNAs were as follows: siLINC2781-1: 5′-CTTCAAATCGGGATGCAAA-3′, siLINC2781-2: 5′-CTGCCAAGAGGCCATTTAA-3′, siLINC2781-3: 5′-CTCCTTTCATCCATTGCTT-3′; siG3BP2#1: 5′-AGTCGAAGCTAAACCAGAA-3′, siG3BP2#2: 5′-CAGGGATATTAGGCGCAAT-3′, siG3BP2#3: 5′-TAAAGCTCCGGAATATTTA-3′; siPIAS1#1: 5′-CUUCAGAGGUUACGAGCAATT-3′, siPIAS1#2: 5′-GGAUCCAGACAGUGAAAUATT-3′, siPIAS1#3: 5′-CAAGAAAGUAGAAGUGAUUTT-3′.

Cells were cultured until 70%–80% confluence and transfected with plasmids using Lipofectamine 3000 (L3000015, ThermoFisher). After 24 h, cells were infected with CVB5 (MOI = 1) and harvested 24 h post-infection (hpi). Mock-infected cells were used as controls.

### RNA isolation and quantitative real-time PCR (qPCR)

Total RNA was extracted from patients’ throat swabs, cells, or tissue using Trizol reagent (15596018, Invitrogen) and validated with the Nano spectrophotometer. Reverse-transcription qPCR (RT-qPCR) was performed using the One Step TB Green PrimeScript Kit (RP066A, Takara). Reactions were carried out using an Applied Biosystems 7500 Real-Time PCR System with the following conditions: 42°C for 5 min, 95 °C for 10 s, and 40 cycles at 95°C for 5 s and 60°C for 34 s. GAPDH or U6 was used as the endogenous reference genes, and the relative RNA levels were calculated using the 2^-ΔΔCt^ method. All primers used are listed in Table S1.

### Cell fraction isolation

Cells were separated into cytoplasmic and nuclear fractions using the PARIS Kit Protein and RNA Isolation System (AM1921, Invitrogen) following the manufacturer’s instructions. Briefly, the cytoplasmic and nuclear fractions were separated by centrifugation, and the nuclear pellets were subsequently lysed. RNA was extracted using a lysis/binding solution, followed by RT-qPCR to detect the expression of LINC2781 in the cytoplasmic fraction (GAPDH was used as the reference gene) and the nuclear fraction (U6 was used as the reference gene).

### Immunoprecipitation and western blotting

Cells were transfected with plasmids after 24 h and lysed with lysis buffer (supplemented with phenyl methane sulfonyl fluoride [PMSF] and phosphatase inhibitor cocktail) at 4°C for 2 h. Then, the collections were incubated with antibody (AE005 or AE003, ABclonal) and protein (A + G) agarose at 4°C for 4 h. The mixtures were washed with phosphate buffer saline (PBS), boiled in loading buffer, and analyzed by western blotting.

The total protein was boiled for 10 min, resolved by SDS-PAGE, and transferred to a PVDF membrane. The membrane was blocked with 5% bovine serum albumin (BSA) in PBS for 2 h, incubated with the primary antibody overnight at 4°C, followed by incubation with the secondary antibody at room temperature for 1 h. The membranes were visualized using enhanced chemiluminescence (ECL) (34577, ThermoFisher). The primary antibodies included Phospho-Stat1 (Tyr701) (58D6) Rabbit mAb (9167, CST), Type I Interferon Induction and Signaling Antibody Sampler Kit (43573, CST), Anti-G3BP2 antibody (ab86135, Abcam), and Anti-CVB5 VP1 (stored in our laboratory).

### Immunofluorescence assay

Cells were transfected with plasmids and infected with CVB5. After 24 h post-infection, the cells were harvested, washed with PBS, fixed with 4% paraformaldehyde for 15 min, and permeabilized with 0.1% Triton X-100 for 10 min at room temperature. Subsequently, samples were blocked with 5% BSA for 2 h, followed by incubation with fluorescently labeled antibody at 4°C for 12 h. Finally, the samples were stained with DAPI and subjected to fluorescent microscopy to visualize the proteins.

### Enzyme-linked immunosorbent assay (ELISA)

Cells were transfected with expression plasmids and infected with CVB5. After 24 h post-infection, the supernatant was analyzed for IFN-I by ELISA Kits, Human IFN-β (#414101, ThermoFisher), and IFN-α (#411001, ThermoFisher), following the manufacturer’s instructions.

### RNA pull-down

Cells were harvested and lysed using base lysis buffer supplemented with 1% protease inhibitor (P1005, Beyotime) and 100 U/mL RNase inhibitor (R0102, Beyotime). Biotin-labeled LINC2781 was transcribed using T7 polymerases (AM1333, ThermoFisher) and purified with the Monarch RNA Cleanup Kit (T2050, New England Biolabs) and then incubated with cell lysates at 37°C for 1 h. The LINC2781 antisense strand was used as a negative control. RNA-protein complexes were recovered using the Pierce Magnetic RNA-Protein Pull-Down Kit (#20164, ThermoFisher). The beads-biotin RNA-protein complexes were washed three times, followed by elution in SDS-PAGE loading buffer and boiling for western blotting analysis.

### RNA immunoprecipitation (RIP) assay

Cells were collected and lysed using an immunoprecipitation lysis buffer (25 mmol/L Tris-HCl, 150 mmol/L NaCl, 1 mmol/L EDTA, 5% glycerol, and 1% NP-40) containing RNase and protease inhibitors. RIP was performed using the Dynabeads Protein A Immunoprecipitation Kit (10006D, ThermoFisher) following the manufacturer’s instructions. Briefly, cells were lysed with a specific antibody, and RNA-protein complexes were subsequently pulled down using protein A/G beads. After several wash steps to remove non-specific binding, the RNA molecules bound to the RNA-binding proteins were isolated and purified. The purified RNA was analyzed by qPCR, and the results were normalized to input RNA levels.

### Construction of G3BP2-knockout cell lines by CRISPR/Cas9

G3BP2 guide RNA sequences, selected using the CRISPR Design Tool (https://www.zlab.bio/guide-design-resources), were inserted into LentiCRISPR v2 vector to construct LentiCRISPR-G3BP2. HEK293T cells were co-transfected with psPAX2, pMD2.G, and LentiCRISPR-G3BP2 plasmids to produce lentivirus targeting endogenous G3BP2. The supernatants were collected 72 h post-transfection, purified with a 0.22 µm filter membrane, and then stored at −80 ˚C. SH-SY5Y cells were infected with lentivirus twice with an interval of 24 h between infections. Then, after multiple passages and puromycin selection, single-cell clones were selected and analyzed by western blotting to identify G3BP2-knockout (G3BP2 KO) cell lines.

### Mouse infection model

BALB/c mice aged 6–8 weeks were purchased from Yunnan University (Kunming, China). To induce the overexpression of LINC2781 in mice, a plasmid encoding LINC2781 was packaged into an adeno-associated virus (AAV) and named AAV2/9-LINC2781 (AAV2/9-Control was used as the control) (NewHelix Biotech, Shanghai, China). BALB/c mice aged 3 days were infected with freshly AAV vectors (5 × 10^11^ genomic copies) via intraperitoneal injection. Fourteen days post-infection, the mice were intraperitoneally challenged via injection with CVB5 (20 LD_50_), and their health status was observed and recorded daily.

All mice were housed in a specific pathogen-free animal facility at the Experimental Animal Center of Kunming University of Science and Technology (Kunming, China), with the approval of the Institutional Animal Care and Treatment Committee of Kunming University of Science and Technology (No. PZWH (Dian) K2023-0044).

### Hematoxylin-eosin (HE) staining

Tissue samples were fixed in 4% formaldehyde for 24 h at room temperature. Subsequently, they were decalcified, dehydrated, and permeabilized using graded alcohol. The tissue was then embedded in paraffin wax, dried, cooled, sectioned, and stained with hematoxylin-eosin. Pathological changes were observed using a light microscope.

### Immunohistochemistry (IHC)

The tissue samples were fixed in 4% formaldehyde, embedded in paraffin, deparaffinized with xylene, and rehydrated with graded alcohol. Endogenous peroxidase of samples was blocked followed by 5% BSA. Slides were incubated with the antibody overnight at 4°C, washed with PBS, and incubated with secondary HRP-conjugated goat anti-IgG at room temperature for 50 min, followed by staining with DAB solution, counterstaining with hematoxylin, dehydrating, and mounting for microscopic examination.

### Clinical subjects

All patients were diagnosed with HFMD or herpetic angina according to the WHO Guide to Clinical Management and Public Health Response. In the case-control study, throat swab samples were obtained from fifty children diagnosed with HFMD or herpetic angina, along with eight healthy controls, at Kunming Children’s Hospital (Kunming, China) between March and June 2024. The healthy controls were age- and sex-matched to the patients and also confirmed to have no other diseases. Informal agreements were obtained from all their legal guardians, and the study was approved by the Medical Ethics Committee of Kunming University of Science and Technology (No. KMUST-MEC-2023-002).

### Statistical analysis

All experiments were carried out three times independently, and the results were presented as mean ± SD. Statistical analysis was performed using the student’s *t*-test in GraphPad Prism9 software. A *P*-value of less than 0.05 was considered statistically significant. Values for *P* ≤ 0.05, *P* ≤ 0.01, and *P* ≤ 0.001 are marked as *, **, and ***, respectively, on the relevant graphs.

### Highlights

LINC2781 promotes the antiviral innate response by activating the JAK-STAT pathway through enhancing the STAT1 activity.

LINC2781 binds to G3BP2 and blocks the G3BP2-STAT1 interaction

G3BP2-mediated the ubiquitination of STAT1 at the tyrosine 701 site

A strong correlation between the expression of LINC2781 and CVB5 infection in cells and clinical samples

## Data Availability

All data generated in the study are included in this published paper.

## SUPPLEMENTAL MATERIAL

Supplemental material. msphere.00062-25-s0001.pdf Supplemental figures and table.

Graphical abstract msphere.00062-25-s0002.pdf Role of lncRNA in virus-host interactions.

ASM does not own the copyrights to Supplemental Material that may be linked to, or accessed through, an article. The authors have granted ASM a non-exclusive, world-wide license to publish the Supplemental Material files. Please contact the corresponding author directly for reuse.
