# Supplementary material for: LINC2781 enhances antiviral immunity against coxsackievirus B5 infection by activating the JAK-STAT pathway and blocking G3BP2-mediated STAT1 degradation
Source: mSphere. 2025 Jul 8;10(7):e00062-25. doi: 10.1128/msphere.00062-25 (PMC12306156; doi:10.1128/msphere.00062-25)
Supplement: Supplemental material. — Supplemental figures and table. [file msphere.00062-25-s0001.pdf]

Supplementary

Figure S1

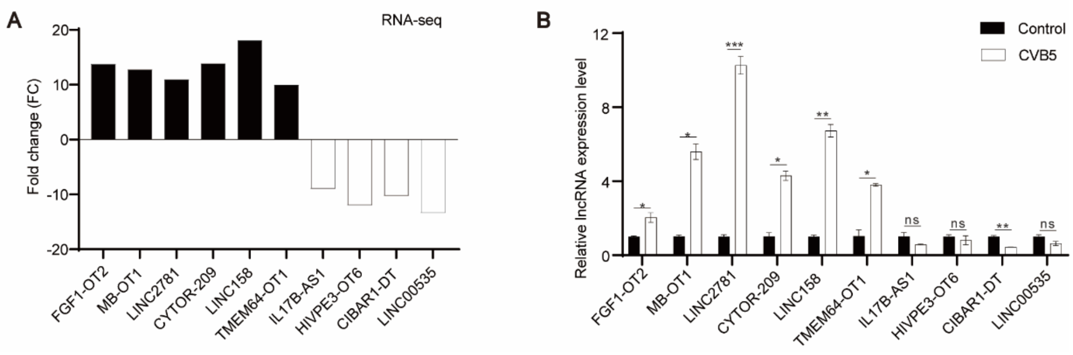

Figure S1. Validation of the expression of lncRNAs following CVB5 infection of SH-SY5Y cells. A. The expression of the top ten differentially expressed lncRNAs were identified from the primary RNA-seq results; **B.** The expression of the top ten differentially expressed lncRNAs were validated using RT-qPCR. Biologically independent experiments (n = 3) were conducted and all data were shown as mean ± SD. Student's t-test was used to detect significant differences, with $P \leq 0.05$ (*), $P \leq 0.01$ (**), $P \leq 0.001$ (***) and ns for no significant difference.

Figure S2

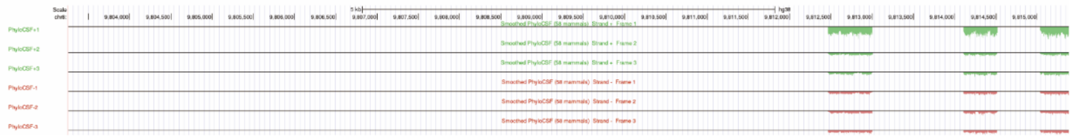

Figure S2. Prediction of LINC2781 protein-coding ability using PhylocSF.

Figure S3

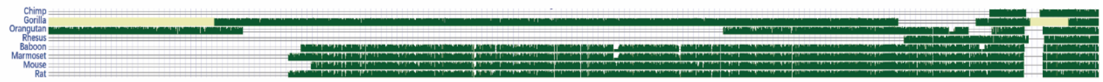

Figure S3. The species homology comparison of LINC2781.

Figure S4

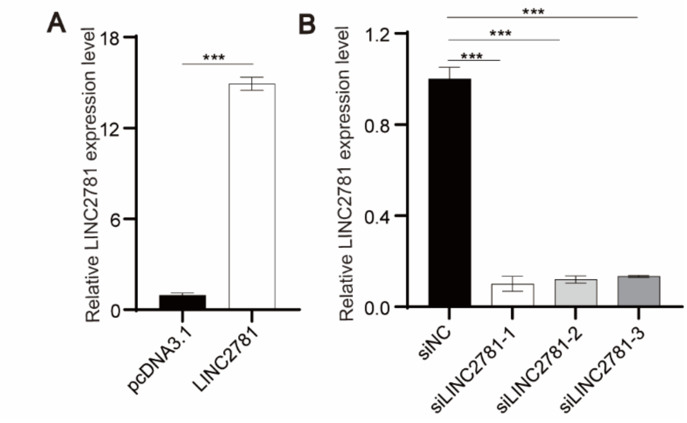

Figure S4. Successful construction of LINC2781 plasmid. A. LINC2781-overexpressing plasmid (LINC2781) or empty vector (pcDNA3.1) was transfected into SH-SY5Y cells for 24 h. The expression of LINC2781 was measured by RT-qPCR; **B.** siLINC2781-1, siLINC2781-2, siLINC2781-3, or empty vector (siNC) was transfected into SH-SY5Y cells for 24 h. The expression of LINC2781 was measured by RT-qPCR. Biologically independent experiments (n = 3) were conducted and all data were shown as mean ± SD. Student's t-test was used to detect significant differences, with P ≤ 0.001 (***).

Figure S5

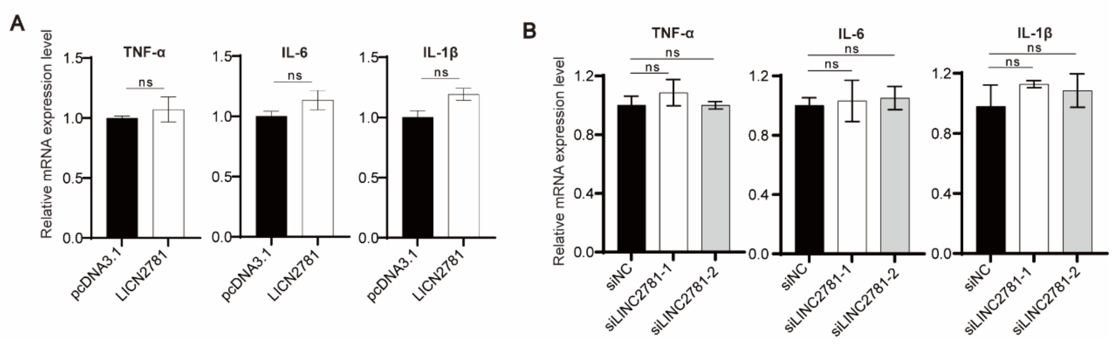

Figure S5. LINC2781 does not affect the expression of pro-inflammatory factors. A. LINC2781-overexpressing plasmid (LINC2781) or empty vector (pcDNA3.1) was

transfected into SH-SY5Y cells, followed by infection with CVB5 (MOI = 1) at 24 h post-transfection. The expression of pro-inflammatory factors was measured by RT-qPCR; **B.** siLINC2781-1, siLINC2781-2, or empty vector (siNC) was transfected into SH-SY5Y cells, followed by infection with CVB5 (MOI = 1) at 24 h post-transfection. The expression of pro-inflammatory factors was measured by RT-qPCR. Biologically independent experiments (n = 3) were conducted and all data were shown as mean ± SD. Student's t-test was used to detect significant differences, with ns for no significant difference.

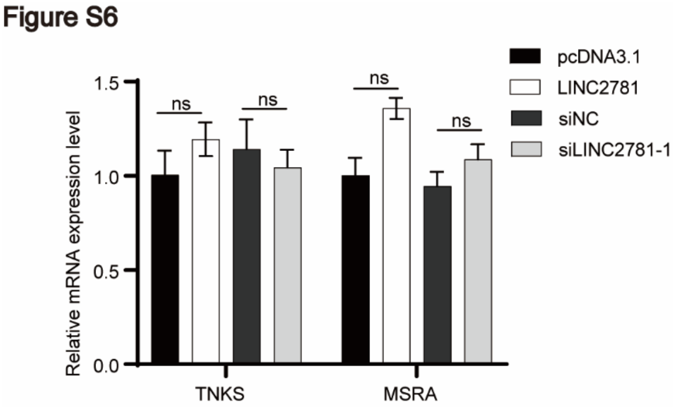

Figure S6. LINC2781 does not affect the expression of the neighboring genes. LINC2781-overexpressing plasmid (LINC2781), siLINC2781-1, or empty vector was transfected into SH-SY5Y cells, followed by infection with CVB5 (MOI = 1) at 24 h post-transfection. The expression of neighboring genes was measured by RT-qPCR. Biologically independent experiments (n = 3) were conducted and all data were shown as mean ± SD. Student's t-test was used to detect significant differences, with ns for no significant difference.

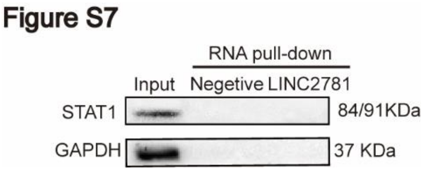

Figure S7. LINC2781 does not bind directly to STAT1 protein. RNA pull-down analysis of LINC2781 binding to STAT1. SH-SY5Y cells were infected with CVB5 (MOI = 1) and harvested at 24 hpi. The biotinylated LINC2781 positive or negative strand was incubated with magnetic beads to obtain protein-RNA complexes. Complexes were then separated by 10% SDS-PAGE gel and analyzed by STAT1 Western blotting.

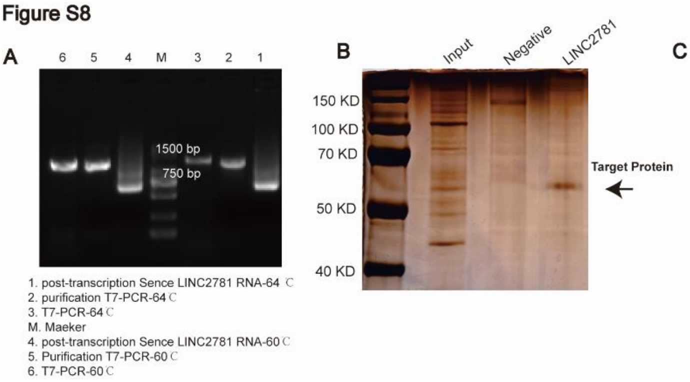

Protein ID	Z-score	Star Rating Score	molecular weight
sp|Q13242|SRSF9_HUMAN	-0.44	2.83	26
sp|Q9NQ94|A1CF_HUMAN	-0.51	2.81	64
sp|P23246|SFPQ_HUMAN	-0.62	2.78	78
sp|O75525|KHDR3_HUMAN	-0.75	2.59	39
sp|Q9UN86|G3BP2_HUMAN	-0.25	2	54
sp|Q9Y2V2|CHSP1_HUMAN	-0.12	2	16
sp|P39019|RS19_HUMAN	-0.27	2	16
sp|Q9NY12|GAR1_HUMAN	-0.41	2	28
sp|Q96C10|DHX58_HUMAN	-0.14	2	77
sp|Q99547|MPH6_HUMAN	-0.27	2	19
sp|Q9BTD8|RBM42_HUMAN	-0.17	2	50
sp|P55795|HNRH2_HUMAN	-0.25	2	49
sp|Q9HC52|CBX8_HUMAN	-0.25	2	43
sp|Q86V81|THOC4_HUMAN	-0.19	2	27
sp|Q15056|IF4H_HUMAN	-0.27	2	27

Figure S8. G3BP2 is identified as a LINC2781-binding protein. A. Verification of T7-LINC2781 probe construction by agarose gel electrophoresis; **B.** Detection of LINC2781-binding proteins by silver staining; **C.** Prediction and scoring of LINC2781-binding proteins.

Figure S9

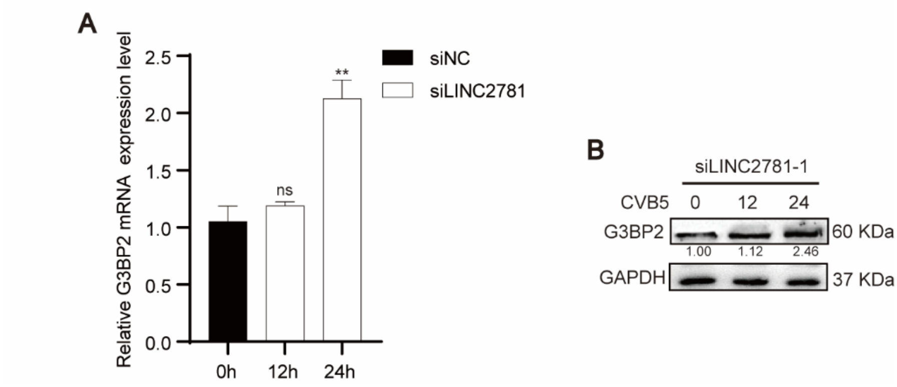

Figure S9. LINC2781 reduces the expression of G3BP2. siLINC2781-1 or empty vector was transfected into SH-SY5Y cells, followed by infection with CVB5 (MOI = 1) at 24 h post-transfection. Cells and supernatants were harvested at 24 hpi. **A.** The expression of G3BP2 mRNA was measured by RT-qPCR; **B.** The expression of the G3BP2 protein was measured by Western blotting. Biologically independent experiments (n = 3) were conducted and all data were shown as mean ± SD. Student's t-test was used to detect significant differences, with P ≤ 0.01 (**) and ns for no significant difference. The band intensity of proteins was quantified and the ratios of the target protein to GAPDH were shown.

Figure S10

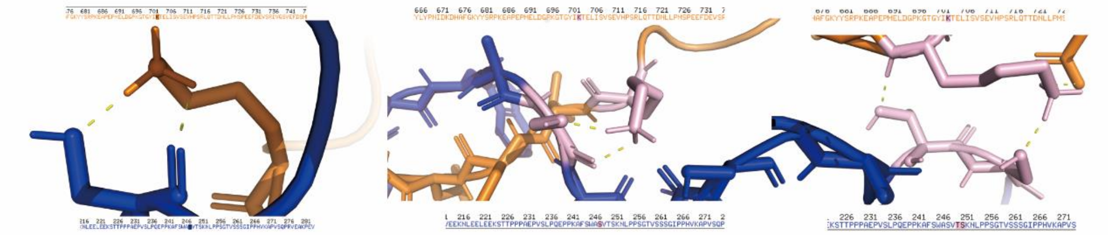

Figure S10. The prediction of STAT1 and G3BP2 binding sites (https://cluspro.bu.edu/queue.php). Yellow indicates STAT1, and blue indicates G3BP2.

Figure S11

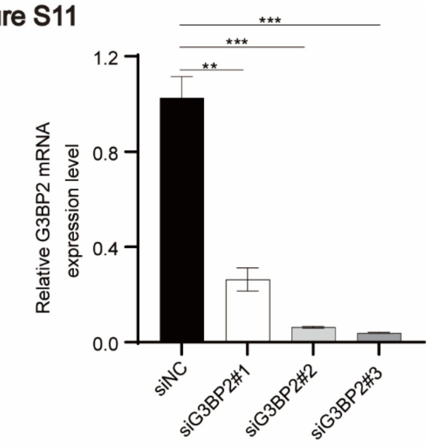

Figure S11. Successful knockdown of G3BP2. siG3BP2#1, siG3BP2#2, siG3BP2#3 or an empty vector (siNC) was transfected into SH-SY5Y cells for 24 h. The expression of G3BP2 mRNA was measured by RT-qPCR. Biologically independent experiments (n = 3) were conducted and all data were shown as mean ± SD. Student's t-test was used to detect significant differences, with P ≤ 0.05 (*), P ≤ 0.01 (**), P ≤ 0.001 (***).

Figure S12

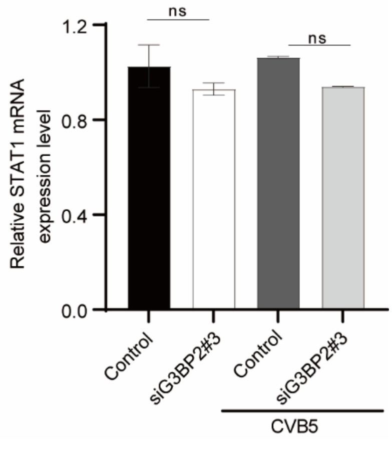

Figure S12. G3BP2 does not affect the expression of STAT1 mRNA. siG3BP2#3 or empty vector was transfected into SH-SY5Y cells, followed by infection with CVB5 (MOI = 1) at 24 h post-transfection. Cells and supernatants were harvested at 24 hpi. The expression of STAT1 mRNA was measured by RT-qPCR. Biologically independent experiments (n = 3) were conducted and all data were shown as mean ± SD. Student's

t-test was used to detect significant differences, with ns for no significant difference.

Figure S13

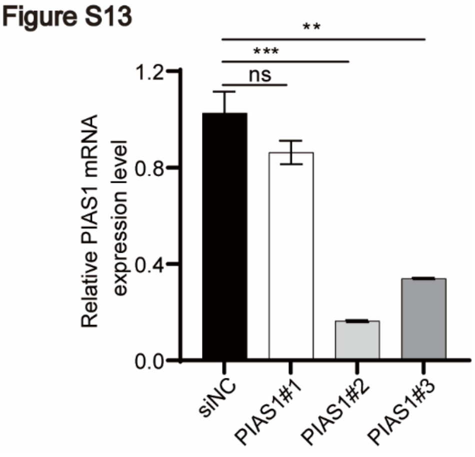

Figure S13. Successful knockdown of PIAS1. siPIAS1#1, si PIAS1#2, si PIAS1#3 or an empty vector (siNC) was transfected into SH-SY5Y cells for 24 h. The expression of PIAS1 mRNA was measured by RT-qPCR. Biologically independent experiments (n = 3) were conducted and all data were shown as mean ± SD. Student's t-test was used to detect significant differences, with $P \leq 0.01$ (**), $P \leq 0.001$ (***) and ns for no significant difference.

Figure S14

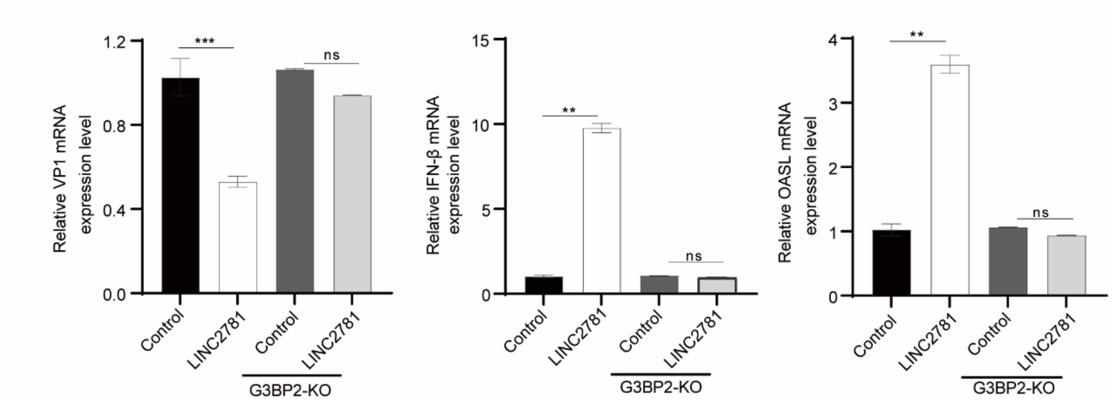

Figure S14. Loss of LINC2781 functionality in G3BP2 knockout cells. LINC2781-overexpressing plasmid (LINC2781) or an empty vector (pcDNA3.1) was transfected

into 293T cells, followed by CVB5 infection CVB5 (MOI = 1) at 24 h post-transfection. Cells and supernatants were harvested at 24 hpi. The expression of CVB5 VP1, IFN-β and OASL mRNA were measured by RT-qPCR. Biologically independent experiments (n = 3) were conducted and all data were shown as mean ± SD. Student's t-test was used to detect significant differences, with P ≤ 0.01 (**), P ≤ 0.001 (***) and ns for no significant difference.

Figure S15

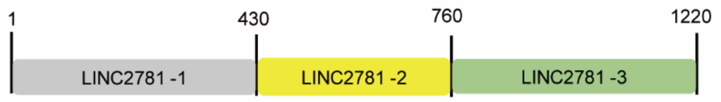

Figure S15. The schematic diagram of the truncated functional domain of LINC2781.

Figure S16

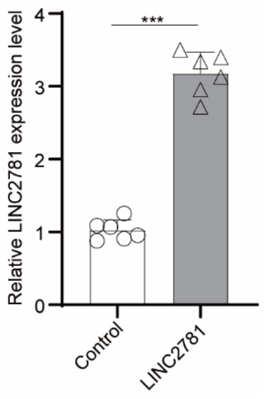

Figure S16. High expression of LINC2781 in mice blood. Fourteen days after AAV2/9-LINC2781 injection, RNA extracted from the blood of mice, followed by RT-PCR amplification to detect the expression of LINC2781. Biologically independent experiments (n = 3) were conducted and all data were shown as mean ± SD. Student's t-test was used to detect significant differences, with P ≤ 0.001 (***).

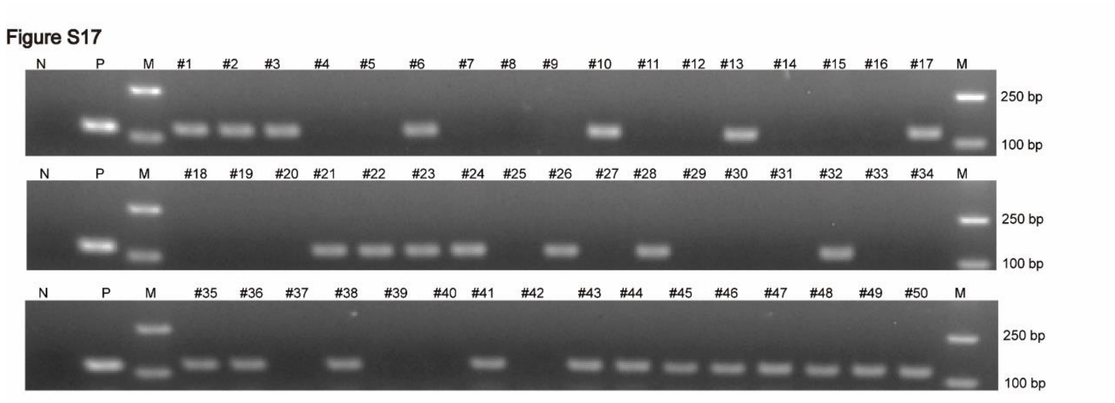

Figure S17. Gel electrophoresis of RT-PCR amplification results of CVB5. RNA extracted from CVB5 (positive control) and patient samples (#1 to #50), followed by RT-PCR amplification to detect the expression of CVB5 VP1. Numbers represent patient samples, P indicates positive controls, N indicates negative controls, and M represents DNA marker (DL2000).

Table S1 Primers used in this study

Primer name	Primer sequence（5’-3’）
GAPDH	F: GAGTCAACGGATTTGGTCGT; R: GACAAGCTTCCCGTTCTCAG
U6	F: CTCGCTTCGGCAGCACA; R: AACGCTTCACGAATTTGCGT
LINC2781	F: AAGAGTGGGAATGCTGCCAA; R: AGCAGGCTCAAGCATCTCAC
LINC2781-1	F: AGTAACTGCTACGCCAGACAA; R: CCCACCACCCTGACTAACTC
LINC2781-2	F: TGTGTTTGCTCCAAGCCTTT; R: TGCTGACGAGAGTCCTGTAG
LINC2781-3	F: GTGTTCAAGTGTGCAACGCA; R: CATCTCACCCATCACACGGG
G3BP2	F: AGGAGATATGGAACAGAATG; R: TTGGTATTGATGCGAAGT
CVB5 VP1	F: GGAGATAGGGTGGCAGATGTAAT; R: GAGAGTGGTGATCGCTGCGCGA
IFN-α2	F: AAGGCTGAAACCATCCCTGT; R: CACCCCCACCCCCTGTATC
IFN-β	F: CTTGGATTCCTACAAAGAAGCAGC; R: TCCTCCTTCTGGAACTGCTGCA
TNF-α	F: GCCACCACGCTCTTCTGTCTAC; R: GGGTCTGGGCCATAGAACTGAT
IL-1β	F: ACCTTCCAGGATGAGGACATGA; R: CTAATGGGAACGTCACACACCA
IL-6	F: CACATGTTCTCTGGGAAATCG; R: TTGTATCTCTGGAAGTTTCAGATTGTT
OASL	F: TTGTGCCTGCCTACAGAGC; R: TTCAGCTTAGTTGGCCGATGT
MXA	F: TTCAGCACCTGATGGCCTATC; R: TGGATGATCAAAGGGATGTGG
ISG15	F: CTCTGAGCATCCTGGTGAGGAA; R: AAGGTCAGCCAGAACAGGTCGT
ISG20	F: TGACCTGAAGCACGACTTCC; R: CAGGCTGTTCTGGATGCTCT
IFIT1	F: TCTCAGAGGAGCCTGGCTAAG; R: CCACACTGTATTTGGTGTCTAGG
IFIT2	F: ACCTCTGGACTGGCAATAGC; R: GTCAGGATTCAGCCGAATGG
IFITM3	F: CATCGTCATCCCAGTGCTGAT; R: ATGGAAGTTGGAGTACGTGGG

128
