# Supplementary material for: LINC2781 enhances antiviral immunity against coxsackievirus B5 infection by activating the JAK-STAT pathway and blocking G3BP2-mediated STAT1 degradation
Source: mSphere. 2025 Jul 8;10(7):e00062-25. doi: 10.1128/msphere.00062-25 (PMC12306156; doi:10.1128/msphere.00062-25)
Supplement: Graphical abstract — Role of lncRNA in virus-host interactions. [file msphere.00062-25-s0002.pdf]

Graphical Abstract

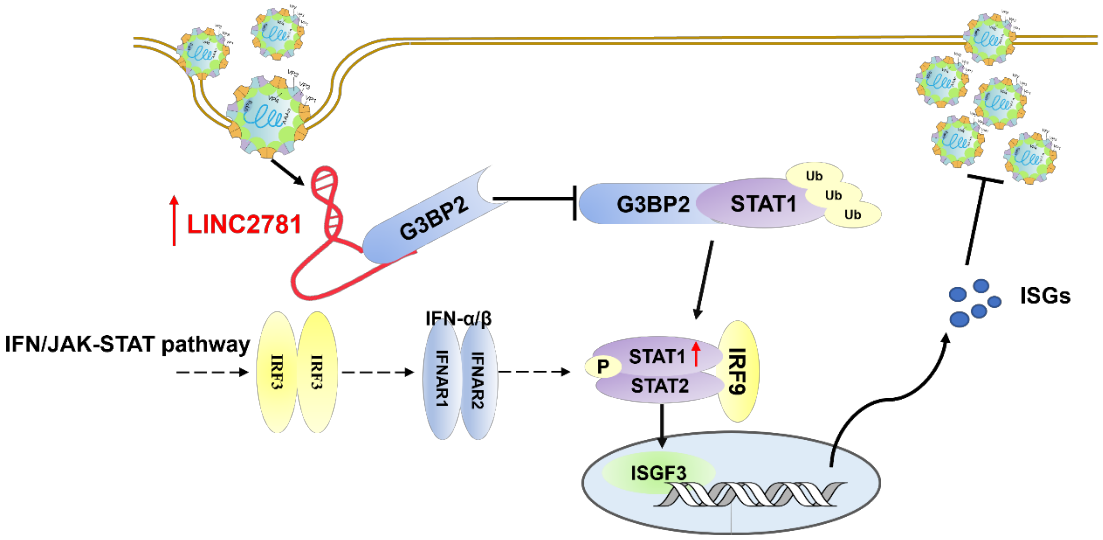

We investigate the role of lncRNA in virus-host interactions and identify a novel cytoplasmic lncRNA, LINC2781, whose expression is upregulated following CVB5 infection. LINC2781 specifically binds to G3BP2, preventing G3BP2 from degrading STAT1, thereby activating the JAK-STAT pathway, promoting the expression of ISGs, and ultimately inhibiting viral replication.
